# Prevalence and determinants of anxiety and depression in adult patients with atopic dermatitis in Tanzania

**DOI:** 10.1093/skinhd/vzag134

**Published:** 2026-08-12

**Authors:** Gloria E Masenga, Elisante J Masenga, Doriane Sabushimike, Kim Madundo, Lulyrita Kini, Jere A Mshana, Muzna Masoud, Janeth Mwase, Nelson Mosha, Aidan Mashuhuri, Agness B Kessy, Sylvia Kilenga, Peter Schmid-Grendelmeier, Alain Taieb, Ousmane Faye, Daudi R Mavura

**Affiliations:** Department of Dermato-venerology, Kilimanjaro Christian Medical Centre, Moshi, Tanzania; Department of Dermato-venerology, Kilimanjaro Christian Medical Centre, Moshi, Tanzania; Kamenge Military Hospital, Bujumbura, Burundi; Department of Psychiatry, Kilimanjaro Christian Medical Centre, Moshi, Tanzania; Department of Dermato-venerology, Kilimanjaro Christian Medical Centre, Moshi, Tanzania; Department of Dermato-venerology, Kilimanjaro Christian Medical Centre, Moshi, Tanzania; Department of Dermato-venerology, Kilimanjaro Christian Medical Centre, Moshi, Tanzania; Department of Dermato-venerology, Kilimanjaro Christian Medical Centre, Moshi, Tanzania; Department of Dermato-venerology, Kilimanjaro Christian Medical Centre, Moshi, Tanzania; Department of Dermato-venerology, Kilimanjaro Christian Medical Centre, Moshi, Tanzania; Department of Dermato-venerology, Kilimanjaro Christian Medical Centre, Moshi, Tanzania; Department of Dermato-venerology, Kilimanjaro Christian Medical Centre, Moshi, Tanzania; University Hospital of Zurich, Allergy Unit, Zurich, Switzerland; INSERM U1312, University of Bordeaux, Bordeaux, France; Hospital of Dermatology, Bamako, Mali; Department of Dermato-venerology, Kilimanjaro Christian Medical Centre, Moshi, Tanzania

## Abstract

**Background:**

Atopic dermatitis (AD) is a chronic inflammatory skin disease increasingly associated with psychiatric co­morbidities, particularly anxiety and depression. Yet, evidence from sub-Saharan Africa remains scarce.

**Objectives:**

To determine the prevalence and associated factors of anxiety and depression among adult patients with AD in Northern Tanzania.

**Methods:**

A hospital-based cross-sectional study was conducted at the Regional Dermatology Training Centre (RDTC) between January 2022 and July 2023. AD was diagnosed using the Hanifin and Rajka criteria, and severity assessed with Scoring Atopic Dermatitis (SCORAD). Anxiety and depression were evaluated using the General Anxiety Disorder-7 and Patient Health Questionnaire-9 tools, respectively. Descriptive statistics and logistic regression were used to identify associations. *P*-values < 0.05 were considered statistically significant.

**Results:**

Overall, 241 adult patients with AD attending RDTC were enrolled, of whom 57.3% (*n* = 138/241) were women. The median age was 38 years (interquartile range 28–56). The prevalences of anxiety and depression were 41.5% and 30.3%, respectively. Anxiety was found to be associated with AD severity and cigarette smoking, while depression was significantly associated with female sex, age 40–59 years, occupation and AD severity.

**Conclusions:**

A considerable proportion of patients with AD in Tanzania experience psychological distress. Routine mental health screening and integrated multidisciplinary care are crucial in AD management, especially in low-resource settings.

## Teaser

This study examines the burden and predictors of anxiety and depression among adults living with atopic dermatitis in Northern Tanzania. Almost half of the patients reported anxiety and nearly one-third reported depressive symptoms, with psychological distress closely linked to disease severity. These findings underscore the importance of incorporating routine mental health assessment and coordinated multidisciplinary care into the management of atopic dermatitis, particularly in resource-limited settings.

What is already known about this topic?

- Atopic dermatitis (AD) is increasingly associated with psychiatric comorbidities such as anxiety and depression.
- This association has most commonly been studied in high-income countries.

What does this study add?

- This study adds to the body of knowledge about psychiatric comorbidities in patients with AD in sub-Saharan Africa.

Atopic dermatitis (AD), also referred to as atopic eczema, is a chronic, relapsing inflammatory skin disorder characterized by intense pruritus, eczematous lesions and a substantial impact on patients’ quality of life.^1^ It results from a complex interplay of genetic susceptibility, epidermal barrier dysfunction, immune dysregulation and environmental exposures.^2^ AD is increasingly recognized as a global health concern due to its rising prevalence and psychosocial burden, especially in low- and middle-income countries.^3^

AD affects approximately 2–10% of adults and up to 10% of ­children in high-income countries.^2^ However, epidemiological data from sub-Saharan Africa remain limited and likely under-­represent the true burden of disease due to misdiagnosis and limited healthcare access. A study conducted in Ethiopia reported an adult ­prevalence of 1.2%, with higher rates in urban regions.^4^ In Tanzania, comprehensive data are scarce, and the disease is often under-recognized or misattributed to infections or traditional illnesses.

Beyond its dermatological manifestations, AD significantly affects psychological wellbeing. The persistent itch, visible lesions, social stigma and frequent sleep disturbances have been linked to elevated rates of anxiety and depression.^5,6^ It has been suggested that the role of systemic inflammation, including cytokine imbalance (e.g. elevated interleukin-4) and dysregulated cortisol levels, may contribute to psychiatric symptoms among patients with AD.^7^

High prevalences of anxiety and depression in patients with AD have been reported in some cohorts, with a possible correlation with disease severity.^7,8^ Nonetheless, information in low-income countries is lacking. These findings underscore the substantial comorbidity between AD and mental health disorders, emphasizing the need for patients to be screened for these conditions. Unfortunately, in most African countries patients have limited access and there is a scarcity of dermatologists.^9^ Dermatologists in resource-limited settings often prioritize the management of skin symptoms, and mental health concerns may remain under-recognized and insufficiently addressed. Many patients do not report their mental health symptoms due to cultural barriers, lack of awareness, stigma and scarcity of mental health specialists.

The aim of this study was to determine the prevalence and associated factors of anxiety and depression in adult patients with AD in Tanzania.

## Patients and methods

### Study design and setting

We conducted a hospital-based cross-sectional study conducted at the Regional Dermatology Training Centre (RDTC) of Kilimanjaro Christian Medical Centre in Moshi, Tanzania. The centre serves as a tertiary referral facility for Northern Tanzania and neighbouring countries.

### Participants

Eligible participants included consenting adults (aged ≥ 18 years) diagnosed with AD between January 2022 and July 2023. AD diagnosis was based on the Hanifin and Rajka criteria. Inclusion criteria were all consenting adult patients with AD attending the RDTC clinic during the study period. Exclusion criteria were (i) patients with pre-existing psychiatric disorders, (ii) patients with other chronic dermatological disease and (iii) patients with cognitive impairment.

### Sample size and sampling

Consecutive sampling was employed, with a minimum sample size of 211 calculated using the Kish and Leslie formula, assuming a 16.5% prevalence of AD from previous RDTC records.

### Data collection

Sociodemographic and clinical information was collected using structured questionnaires, and AD severity was evaluated with a validated tool, Scoring Atopic Dermatitis (SCORAD).^10^ SCORAD scores < 25 were considered to represent mild AD, 25–50 moderate AD and > 50 severe AD.

Anxiety and depression were measured with the validated and reliable Swahili versions of the General Anxiety Disorder (GAD)-7 and Patient Health Questionnaire (PHQ)-9, ensuring appropriateness for our setting.^11^ GAD-7 scores of 0–4 signify minimal to no anxiety, 5–9 mild anxiety, 10–14 moderate anxiety and 15–21 severe anxiety. PHQ-9 scores of 0–4 imply minimal to no depression, 5–9 mild depression, 10–14 moderate depression, 15–19 moderately severe depression and 20–27 severe depression. For participants who were unable to read or write, interviews were administered by the principal investigator. Participants with moderate-to-severe psychological symptoms were referred for psychiatric evaluation.

### Statistical analysis

Data were entered into and analysed with SPSS v22 (IBM, Armonk, NY, USA). Descriptive statistics were used to summarize baseline characteristics. Bivariate associations were assessed using χ^2^ tests, and multivariable logistic regression was used to identify independent predictors of anxiety and depression.

## Results

### Participant characteristics

Among 1050 patients presenting to RDTC with skin conditions, 280 met the screening criteria for AD. Of these, 241 fulfilled the diagnostic criteria and consented to participate, giving a proportion of 23% with AD within the clinic population (Figure 1).

**Figure 1:**
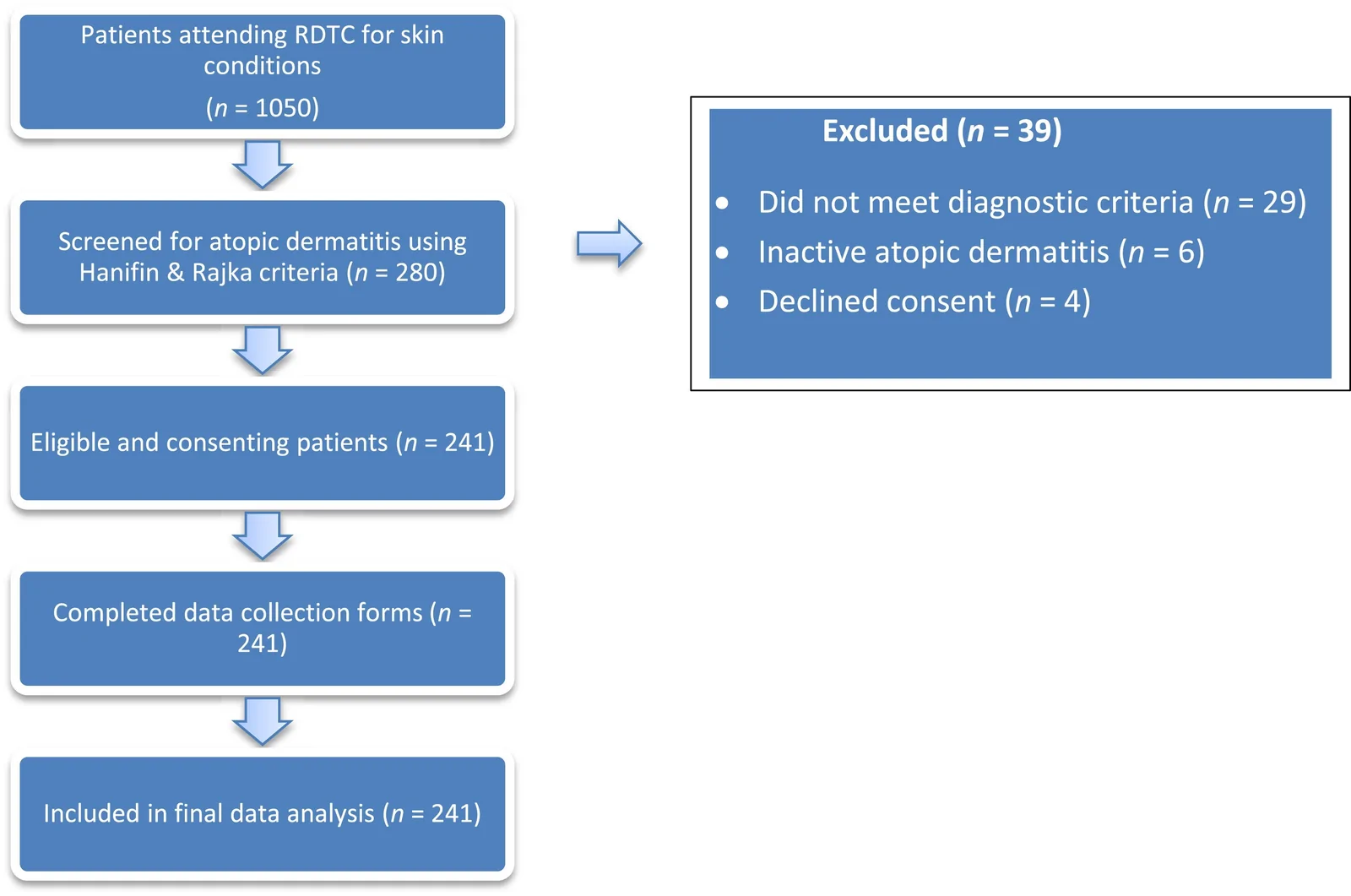
Patient enrolment flowchart. RDTC, Regional Dermatology Training Centre.

Among the 241 adults with AD, women comprised the majority (*n* = 138/241; 57.3%), while men accounted for 42.7% (*n* = 103/241). The median age was 38 years [interquartile range (IQR) 28–56]. More than half of the participants lived in urban areas (*n* = 133/241; 55.2%), and nearly all had received formal education (*n* = 229/241; 95.0%).

Regarding disease severity, 43.2% (*n* = 104/241) of participants had mild AD, while 37.3% (*n* = 90/241) experienced moderate AD and 19.5% (*n* = 47/241) had severe disease according to the SCORAD index. The median duration of AD was 6 years (IQR 2–15).

Lifestyle and comorbidity factors were also notable. Over half of the participants were obese (*n* = 129/241; 53.5%). Cigarette smoking was reported by 19.1% (*n* = 46/241) and alcohol use by 46.9% (*n* = 113/241).

### Prevalence and associated factors of anxiety and depression in atopic dermatitis

Overall, 41.5% (*n* = 100/241) of participants reported symptoms of anxiety, with 32.4% (*n* = 78/241) classified as mild, 7.9% (*n* = 19/241) as moderate and 1.2% (*n* = 3/241) as severe. A positive association was found between AD severity and anxiety. Participants with severe AD had over 12 times higher odds of anxiety than those with mild disease [adjusted odds ratio (aOR) 12.1, 95% confidence interval (CI) 4.66–31.7; *P* < 0.01]. Moderate AD was associated with three times higher odds (aOR 3.17, 95% CI 1.51–6.65; *P* < 0.01). Cigarette smoking was also positively associated with anxiety, with patients who smoked having more than three times the odds of those who did not (aOR 3.19, 95% CI 1.34–7.58; *P* = 0.01) (Table 1).

**Table 1:** Adjusted analysis of factors associated with anxiety and depression among patients with atopic dermatitis (*n* = 241)

Variable	Anxiety		Depression	
	aOR (95% CI)	*P*-value	aOR (95% CI)	*P*-value
Age (years)				
18–39	Reference		Reference	
40–59	1.04 (0.45–2.41)	0.93	0.37 (0.15–0.90)	0.03
60–100	1.10 (0.39–3.14)	0.86	0.90 (0.33–2.44)	0.84
Sex				
Male	Reference		Reference	
Female	1.04 (0.52–2.09)	0.91	2.61 (1.28–5.33)	0.01
Education level				
Primary or less	Reference		Reference	
Secondary	2.56 (0.90–7.26)	0.08	0.40 (0.14–1.15)	0.09
Diploma	1.39 (0.39–5.01)	0.61	0.13 (0.04–0.49)	0.06
Degree or higher	1.83 (0.51–6.59)	1.83	0.32 (0.09–1.08)	0.07
Occupation				
None	Reference		Reference	
Student	0.62 (0.08–4.80)	0.65	5.07 (0.56–46.3)	0.15
Employed	0.45 (0.09–2.15)	0.32	8.07 (1.53–42.5)	0.01
Self-employed	0.84 (0.22–3.14)	0.79	4.29 (1.13–16.3)	0.03
Alcohol intake				
Yes	2.00 (0.99–4.04)	0.05	1.50 (0.74–3.03)	0.26
No	Reference		Reference	
Cigarette smoking				
Yes	3.19 (1.34–7.58)	0.01	1.84 (0.95–3.56)	0.01
No	Reference		Reference	
SCORAD score				
Mild	Reference		Reference	
Moderate	3.17 (1.51–6.65)	< 0.01	1.79 (0.84–3.80)	0.13
Severe	12.1 (4.66–31.7)	< 0.01	4.18 (1.71–10.2)	< 0.01

aOR, adjusted odds ratio; CI, confidence interval; SCORAD, Scoring Atopic Dermatitis.

Depression was observed in 30.3% (*n* = 73/241) of participants, with 23.7% (*n* = 57/241) experiencing mild symptoms, 5.8% (*n* = 14/241) moderate and 0.8% (*n* = 2/241) moderately severe to severe. Women were more likely to experience depression, with over twice the odds compared with men (aOR 2.61, 95% CI 1.28–5.33; *P* = 0.01). Employment status showed a strong positive association: employed participants had eight times higher odds of depression (aOR 8.07, 95% CI 1.53–42.5; *P* = 0.01), and self-­employed respondents had four times higher odds (aOR 4.29, 95% CI 1.13–16.3; *P* = 0.03) than those who were not employed.

Despite a majority of our participants having obesity, with over twice the odds of depression than those who were not (aOR 2.11, 95% CI 0.50–8.80), this was not statistically significant. A positive correlation was also observed between AD severity and depression, with severe disease remaining the strongest predictor, conferring over four times higher odds compared with mild AD (aOR 4.18, 95% CI 1.71–10.2; *P* < 0.01) (Table 1).

## Discussion

This study highlights a substantial burden of psychological distress among adults with AD in Northern Tanzania, with anxiety affecting over two-fifths and depression nearly one-third of participants. These findings reinforce the growing recognition that AD extends well beyond the skin, carrying important mental health consequences that are often underappreciated in routine clinical care, particularly in resource-limited settings.

The prevalence observed in this cohort is consistent with findings from dermatology-focused literature. A recent systematic review and meta-analysis reported significantly increased risks of anxiety, depression and severe mental illness among individuals with AD and psoriasis, underscoring the close relationship between chronic inflammatory skin disease and psychiatric comorbidity.^12^ Similarly, global estimates suggest that anxiety and depression affect a substantial proportion of patients with chronic skin disease, with figures broadly comparable with those reported here.^13^ Taken together, these data suggest that the psychological burden observed in this Tanzanian cohort is not an isolated finding, but part of a wider, global pattern.

Importantly, evidence from populations with skin of colour and low- and middle-income settings mirrors these observations. In Ethiopia, high levels of anxiety have been documented among patients with chronic dermatological conditions, highlighting the psychosocial impact of skin disease within African contexts.^14^ Comparable findings have been reported in South East Asia, where patients with AD in tertiary-care settings experience similarly elevated levels of anxiety and depression.^15^ In addition, a recent scoping review from sub-Saharan Africa emphasized the profound impairment of quality of life associated with AD, particularly among younger populations, pointing to the long-term psychosocial implications of the disease across the life course.^16^ These parallels strengthen the external validity of our findings and underline the need to consider cultural and health system factors when addressing mental health in dermatology.

Disease severity emerged as a key determinant of both anxiety and depression in this study. Patients with more severe AD were significantly more likely to experience psychological distress, a finding that aligns with previous work demonstrating that persistent itch, sleep disruption and visible skin changes can cumulatively impair emotional wellbeing.^17^ The relationship is likely to be bidirectional: worsening skin disease contributes to psychological distress, which in turn may exacerbate inflammatory pathways and disease activity. This interplay highlights the importance of integrated care approaches that address both dermatological and psychological dimensions of AD.

Sex differences were also evident, with women more likely to report depressive symptoms. This is in keeping with prior studies showing higher levels of anxiety and depression among women with AD,^18^ and may reflect a combination of biological susceptibility and greater psychosocial impact of visible skin disease. Broader population-based data further suggest that mental health outcomes in AD are shaped by intersecting factors including ethnicity, sex and social context,^19^ which may be particularly relevant in diverse and rapidly changing societies.

The association between cigarette smoking and anxiety observed in this cohort is also noteworthy. Smoking may serve as a coping strategy for psychological distress, while simultaneously contributing to systemic inflammation and worsening of skin symptoms, thereby reinforcing a harmful cycle. Similarly, the observed relationship between occupational status and depression suggests that the challenges of managing a chronic, visible condition within the workplace may amplify psychological strain. Previous studies have shown that AD can negatively affect work productivity, increase absenteeism and limit career opportunities, all of which may contribute to emotional distress.^12^

Taken together, these findings emphasize that AD should be approached as a multidimensional condition requiring holistic management. In many African settings, mental health remains under-recognized, and patients may be reluctant to report psychological symptoms due to stigma or limited awareness. Integrating routine mental health screening into dermatology services, even through brief, validated tools, could help identify patients in need of further support and improve overall outcomes. Commonly used tools include the GAD-7 and PHQ-9,^11^ which were used in this study. Dermatologically focused tools include the Dermatology Life Quality Index^20^ and the one-question Distress Thermometer.^21^ The integration of such tools into routine care may support earlier recognition and more holistic management of AD.

This study has several important limitations that should be considered when interpreting the findings. Firstly, the hospital-based nature of the sample may limit generalizability, as participants attending a tertiary dermatology centre may represent more severe or complex cases. Secondly, the study was conducted at a single centre, which may not fully capture the diversity of patients across different regions of Tanzania or sub-Saharan Africa. Thirdly, the cross-sectional design precludes conclusions about the causality of AD severity and psychological outcomes. In addition, the use of a clinic-based sample introduces the possibility of selection bias, as individuals seeking care may differ systematically from those in the community. Finally, although multivariable analysis was performed, the potential for residual confounding cannot be excluded.

Despite these limitations, the study also has notable strengths. It addresses an important and relatively understudied topic within the African context, where data on the mental health burden of AD remain scarce. The sample size is robust for a single-­centre study and allows for meaningful analysis of associated factors. The use of validated and widely accepted tools for assessing anxiety, depression and disease severity (PHQ-9, GAD-7 and SCORAD) strengthens the reliability and comparability of the findings. Furthermore, the identification of clinically relevant associations, particularly the role of disease severity and modifiable factors such as smoking, provides practical insights that can inform patient management and future research.

In conclusion, despite cultural differences across the world, anxiety and depression are quite common in African patients with AD. Therefore, integrating systematic psychological screening into routine dermatological care and developing a specific evaluation tool for early detection are appealing.

## Data Availability

The data that support the findings of this study are available from the corresponding author upon reasonable request.
